# Swyer Syndrome: A diagnostic challenge

**DOI:** 10.5935/1518-0557.20240096

**Published:** 2025

**Authors:** Imen Bannour, Badra Bannour, Salma Ferjani, Sassi Boughizane

**Affiliations:** 1 Department of Gynecology and Obstetrics, University Hospital Farhat Hached, Faculty of Medicine, Ibn Al Jazzar, University of Sousse, Sousse, Tunisia

**Keywords:** Swyer syndrome, 46XY, gonadal dysgenesis, uterus, primary amenorrhea

## Abstract

Swyer syndrome, represents a rare manifestation of primary amenorrhea arising from gonadal dysgenesis. This syndrome is distinguished by the manifestation of a female phenotype despite a 46, XY karyotype. We present the case of a patient aged 32 the second of three sisters; consulted for the first time with a main complaint of primary unexplored amenorrhea responsible for infertility of 1 year with a female phenotype and a male karyotype: 46XY. The laparoscopy performed revealed the presence of a small uterus (an unexpected finding for a feminizing testicular syndrome). The other sisters were respectively examined and found to have the same pathology as their sister and were eventually programmed to have a laparoscopy. 46XY pure gonadal dysgenesis, commonly known as Swyer syndrome, presents as a rare disorder in sexual development. Despite having a 46XY karyotype, affected individuals exhibit a female phenotype. The underlying cause is believed to stem from mutations and deletions affecting the Sex Determining Region Y (SRY) gene located on the short arm of the Y chromosome. Swyer syndrome should be considered in cases of primary amenorrhea with the presence of a uterus. Chromosomal analysis is essential for confirming the diagnosis.

## INTRODUCTION

Swyer syndrome, initially identified by Gim Swyer in 1955, represents a rare manifestation of primary amenorrhea arising from gonadal dysgenesis. This syndrome is distinguished by the manifestation of a female phenotype despite a 46, XY karyotype. Typically, the gonads are either absent or present as streak gonads, while the uterus and fallopian tubes are commonly absent or underdeveloped (King & Conway, 2014). Individuals with Swyer syndrome face an elevated susceptibility to gonadal tumors, notably gonadoblastoma, and may encounter a delay in the onset of puberty (Michala *et al.*, 2008). We present the case of three sisters presenting Swyer.

## CASE REPORT

We present the case of a patient aged 32 the second of three sisters; an older sister aged 34 and a younger sister aged 18. The middleborn sister consulted for the first time with a main complaint of primary unexplored amenorrhea responsible for infertility of 1 year. The medical history revealed that her two sisters also suffer from primary amenorrhea.

Physical examination revealed normal female phenotype with normal breast development and normal female external genitalia. A pelvic ultrasonography revealed the presence of the small uterus and the absence of normal ovaries. A hormonal study was conducted, which showed highly elevated serum concentration of luteinizing hormone and follicle-stimulating hormone with low circulating levels of gonadal steroids (estradiol and testosterone). Testosterone: 0.02 ng/mL (normal values are between 0.05-0.4), estradiol: 7pg/mL (normal values are between 11.3-43.2), luteinizing hormone (LH): 38mIU/mL (normal values are between 1.7-8.6), follicle stimulating hormone: 36,7mIU/mL (normal values are between 1.5-12.4). The hormonal study represented primary hypogonadism and a chromosomal study revealed a male karyotype 46XY.

These findings concluded to the diagnosis of Swyer syndrome. Thus, the patient was scheduled for bilateral gonadectomy due to the risk of developing gonadal tumors.

**Figure 1:**
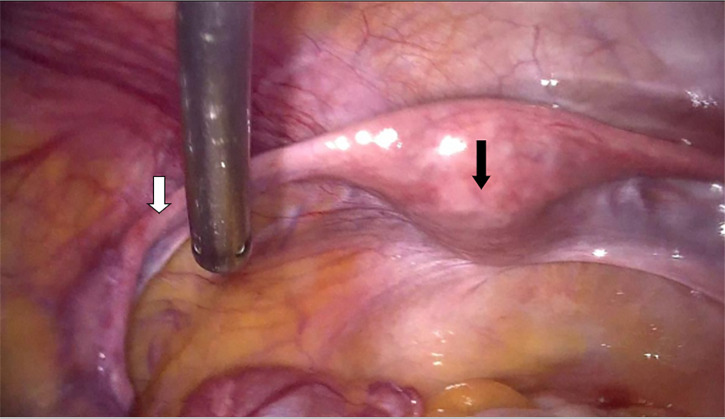
The uterus is often small in size, allowing a wide view of the cul-de-sac of Douglas. Both fallopian tubes are abnormally long. White arrow indicates the abnormally long fallopian tube; black arrow indicates the small uterus.

**Figure 2:**
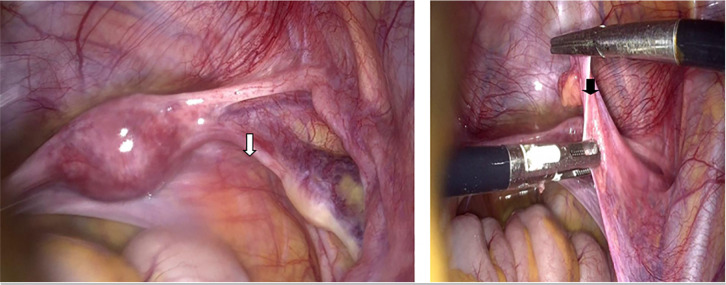
The utero-ovarian ligaments are well individualized, abnormally long and thin. The ovary is reduced to a strip spread over 3 to 4 cm.

The laparoscopy performed revealed a small uterus allowing a wide view of the cul-de-sac of Douglas. Both fallopian tubes are abnormally long, and the utero-ovarian ligaments are well-defined but unusually long and thin. The lombo-ovarian ligament is also less developed. The gonads appear as bands measuring 3 cm (Figures 1 and 2).

The other sisters were thoroughly examined and diagnosed with the same pathology (Swyer syndrome).

## DISCUSSION

46XY pure gonadal dysgenesis, commonly known as Swyer syndrome, presents as a rare disorder in sexual development. Despite having a 46XY karyotype, affected individuals exhibit a female phenotype. The underlying cause is believed to stem from mutations and deletions affecting the Sex Determining Region Y (SRY) gene located on the short arm of the Y chromosome. This genetic aberration is identified in approximately 20% of patients with 46XY pure gonadal dysgenesis (Behtash & Karimi Zarchi, 2007; Kulathilake & Jayasundara, 2015).

### Sex development disorders pathogenesis

The differentiation of testes or ovaries from the common gonadal primordia is regulated by intricate male-specific and female-specific molecular networks involving gene expression, dosage, and interaction.

In normal sex development, the expression of the sex-determining region Y (SRY) gene on the Y chromosome initiates a genetic cascade that directs the undifferentiated gonad to develop into a testis. In the developing testis, Sertoli cells secrete anti-Müllerian hormone (AMH), which induces the regression of the Müllerian ducts. Concurrently, testosterone secretion from Leydig cells facilitates the differentiation of the Wolffian ducts into the seminal vesicles, vas deferens, and epididymis (Ono & Harley, 2013).

The absence of functional SRY gene activity leads to the failure in testosterone and anti-Müllerian hormone production, thereby allowing the embryonic structures derived from the Müllerian duct (paramesonephric duct) to develop. Consequently, individuals with Swyer syndrome develop a uterus, bilateral fallopian tubes, and a normal vagina. However, due to the dysfunctional SRY gene, the Wolffian ducts (mesonephric ducts) fail to develop, resulting in the absence of testes formation. As a result, affected individuals exhibit typical female external genitalia and androgen levels (Patnayak *et al.*, 2012).

Elevated levels of gonadotropin hormones are accompanied by decreased levels of estradiol. This hormonal pattern of hypergonadotropic hypogonadism was observed in our patient. The underdevelopment of the uterus is typically attributed to estrogen deficiency.

### Diagnosis of Swyer syndrome

Individuals with Swyer syndrome are phenotypically female, exhibiting an unambiguously female genital appearance at birth and normal Müllerian structures (Michala *et al.*, 2008). Due to the lack of hormonal activity in the gonads, the condition typically presents during adolescence with primary amenorrhea and delayed puberty. A distinguishing feature of women with Swyer syndrome is their increased adult height that may be attributed to the influence of the Y chromosome or delayed epiphyseal closure due to low sex steroid levels.

Early diagnosis is generally feasible only if a karyotype is performed for other reasons, such as prenatal screening for aneuploidy or as part of familial screening following the diagnosis of a sibling with the condition. However, the vast majority of individuals do not have a family history, with only 4% having an affected sibling in clinical populations.

The initial investigations, should include measurements of serum electrolytes, luteinizing hormone, follicle-stimulating hormone, prolactin, thyroid-stimulating hormone, free thyroid hormone, sex hormone-binding globulin, androstenedione, estradiol, and testosterone. Anti-Müllerian hormone (AMH) and inhibin measurements are optional if there is a question about gonadal status, but they generally do not provide additional information if serum follicle-stimulating hormone levels are elevated. For every person presenting with delayed puberty, a regular peripheral blood karyotype should be requested.

When gonadal dysgenesis is confirmed, tumor markers such as alpha-fetoprotein, beta human chorionic gonadotropin, lactate dehydrogenase, and placental alkaline phosphatase should be considered. Although these tests are often delayed until a gonadal mass is identified, confirming tumor marker status before any surgical procedures can be beneficial (Capito *et al.*, 2011; McCann-Crosby *et al.*, 2014).

Sequencing of specific causative genes is not widely accessible, and the benefit of identifying specific causative genes remains unproven. An exception is the NR5A1 gene, particularly in individuals with an unusual family history, as the pattern of inheritance is unpredictable, making it useful for genetic counseling. However, NR5A1 mutations are likely rare in cases of complete gonadal dysgenesis without a family history, so routine screening for this phenotype may have limited value (Lin *et al.*, 2007).

Patients with Swyer syndrome typically exhibit low levels of androgens and androgen precursors, elevated gonadotropins, low or undetectable AMH, and a non-mosaic 46,XY karyotype on cytogenetic analysis. Adrenal function is usually normal unless the underlying defect involves SF-1 or related adrenal or gonadal factors (Ahmed *et al.*, 2011). The first-line imaging modality is transabdominal pelvic ultrasound, conducted by a sonographer experienced in adolescent anatomy. MRI should be reserved for cases where ultrasonography fails to clearly delineate the relationship of the Müllerian structures or if there are abnormalities in the urinary tract (Ahmed *et al.*, 2011).

In Swyer syndrome, the typical imaging findings include intra-abdominal streak gonads and present Müllerian structures due to impaired AMH secretion during early fetal development. During laparoscopy, the uterus is often small, allowing a wide view of the cul-de-sac of Douglas. Both fallopian tubes are abnormally long, and the utero-ovarian ligaments are well-defined but unusually long and thin. The lombo-ovarian ligament is also less developed. The gonads appear as bands measuring 3 to 4 cm, sometimes showing a small central swelling and increased hilar vascularization, raising concerns about the risk of malignant degeneration.

### Differential diagnoses

The differential diagnoses of Swyer syndrome include complete androgen insensitivity syndrome (CAIS) and Mayer-Rokitansky-Küster-Hauser (MRKH) syndrome. Patients with CAIS exhibit a female phenotype and normal breast development, with morphologically normal testes (which may be undescended) and no Müllerian structures. The absence of the uterus is also being increasingly considered as a diagnostic criterion for CAIS (Da Silva Rios *et al.*, 2015).

MRKH syndrome, another cause of primary amenorrhea, affects approximately 1 in 5000 live female births. This syndrome is characterized by varying degrees of Müllerian duct and vaginal aplasia, along with a rudimentary uterus. However, individuals with MRKH syndrome have normal female sexual characteristics and genotype.

### Follow-up of patients with Swyer syndrome

Patients with dysgenetic gonads, such as those with Swyer syndrome, require meticulous follow-up due to a significant risk of neoplasia. Gonadal tumors develop in approximately 30% of cases, with some studies reporting tumors in up to 75% of patients (Behtash & Karimi Zarchi, 2007; Zieliñska *et al.*, 2007; Kulathilake & Jayasundara, 2015). Most individuals are at risk of developing gonadoblastoma, a benign germ cell and sex cord stromal tumor, which has a substantial potential to progress to dysgerminoma in up to 50% of cases (Hanlon & Kimble, 2015). Dysgerminoma is the most common malignant germ cell tumor type, and it can occur bilaterally in 15% of cases (Zieliñska *et al.*, 2007).

Occasionally, patients present later in life with symptoms such as pelvic pain or a pelvic mass and are found to have dysgerminoma before being diagnosed with Swyer syndrome (Behtash & Karimi Zarchi, 2007). The current recommendation for patients diagnosed with Swyer syndrome is to undergo bilateral gonadectomy to reduce the likelihood of developing gonadal tumors in the future. Laparoscopy is the preferred surgical approach, offering a minimally invasive method for the removal of the gonads (Malhotra *et al.*, 2015). Numerous cases in the literature emphasize the importance of prophylactic and therapeutic bilateral gonadectomy in this patient cohort due to the increased risk of malignant transformation (Hanlon & Kimble, 2015).

The literature clearly supports the need for prophylactic gonadectomies in these patients, but there is no concurrent recommendation for the removal of the fallopian tubes (Zieliñska *et al.*, 2007; Hanlon & Kimble, 2015).

### Management

The management of Swyer syndrome necessitates collaboration among various specialists, including pediatricians, endocrinologists, urologists, psychiatrists, and other healthcare providers. Treatment typically involves estrogen replacement therapy, supplemented with progestin within one to two years to induce secondary sexual characteristics, stimulate pubertal growth, and promote optimal bone health. Early initiation of hormone therapy is crucial to maximize the potential for normal pubertal development. However, there’s no proven benefit of cyclic progesterone supplementation in females lacking a uterus (Wisniewski *et al.*, 2019).

Living with Swyer syndrome can pose significant challenges to self-image and identity, highlighting the importance of counseling and support groups to address emotional and psychological concerns, including gender identity and role determination (Wisniewski *et al.*, 2019).

## CONCLUSION

In summary, Swyer syndrome should be included in the differential diagnosis of primary amenorrhea when imaging studies reveal an absence of visible uterus and ovaries. Chromosomal analysis is essential for confirming the diagnosis, while prompt initiation of hormone replacement therapy is vital for inducing secondary sexual characteristics and averting osteoporosis. Regular screening for gonadal tumors is advised due to the heightened risk of malignancy in those affected. These therapeutic interventions should be complemented with emotional support and psychiatric care.
